# Reg family proteins contribute to inflammation and pancreatic stellate cells activation in chronic pancreatitis

**DOI:** 10.1038/s41598-023-39178-3

**Published:** 2023-07-27

**Authors:** Wenting Chen, Mai Imasaka, Miyu Lee, Hirokazu Fukui, Hiroshi Nishiura, Masaki Ohmuraya

**Affiliations:** 1 Department of Genetics, Hyogo Medical University, 1-1, Mukogawa-Cho, Nishinomiya, Hyogo 663-8501 Japan; 2 Division of Gastroenterology and Hepatology, Department of Internal Medicine, Hyogo Medical University, Nishinomiya, Hyogo 663-8501 Japan; 3 Division of Functional Pathology, Department of Pathology, Hyogo Medical University, Nishinomiya, Hyogo 663-8501 Japan; 4 Present Address: Clinical Training Center, Osaka Medical and Pharmaceutical University, Takatsuki, Osaka 569-8686 Japan

**Keywords:** Chronic pancreatitis, Gene expression

## Abstract

Chronic pancreatitis (CP) is a disease characterized by the inflammation and destruction of pancreatic tissue, leading to the replacement of functional tissue with fibrotic tissue. The regenerating gene (*Reg*) family proteins have recently been implicated in the repair and regeneration of inflamed pancreatic tissue, though the exact mechanisms of their involvement in the pathogenesis of CP are not yet fully understood. To investigate the role of Reg family proteins in CP, we generated global knockout mice (*Reg*^*−/−*^) for *Reg1-3* (*Reg1,2,3a,3b,3d,3g*) genes using the CRISPR/Cas9 system. We then investigated the effect of Reg family protein deficiency in a genetic model of CP (X-SPINK1) mice by knocking out *Reg1-3* genes. We examined pancreatic morphology, inflammatory cytokines expression, and activation of pancreatic stellate cells (PSCs) at different ages. *Reg*^*−/−*^ mice showed no abnormalities in general growth and pancreas development. Deficiency of *Reg1-3* in CP mice led to a reduction in pancreatic parenchymal loss, decreased deposition of collagen, and reduced expression of proinflammatory cytokines. Additionally, Reg proteins were found to stimulate PSCs activation. Overall, our study suggests that *Reg1-3* deficiency can lead to the remission of CP and Reg family proteins could be a potential therapeutic target for the treatment of CP.

## Introduction

Chronic pancreatitis (CP) is a progressive inflammatory disease characterized by pancreatic exocrine insufficiency, fibrosis, and irreversible damage to the pancreas. Despite its high prevalence, the underlying pathophysiology of CP is not yet fully understood, and there are currently no effective treatments for this disease. As it frequently causes digestive and absorption disorders and diabetes due to the deterioration of pancreatic exocrine and endocrine functions, making it a major public health concern^1^.

The regenerating gene (*Reg*) family comprises four groups (*Reg1-4*) with a C-type-like lectin domain. In rodents, Reg family proteins consist of Reg1, Reg2, Reg3a, Reg3b, Reg3g, Reg3d, and Reg4 while in humans, they are REG1A, REG1B, REG3A, REG3G, and REG4^2^. These proteins are expressed in various mammalian organs and have multifunctional effects, including trophic, antiapoptotic, anti-inflammatory, antifibrogenic, antimicrobial, and immunoregulatory effects^3,4^. However, the regulation of expression, exact functional roles, and the underlying mechanism of *Reg* remain incompletely understood.

Our previous microarray analysis of a genetic model of CP mice (X-SPINK1 mice) showed that *Reg* family expression was significantly increased during the onset of the disease, suggesting that it may be involved in pancreatic fibrosis. [dataset] (https://www.ncbi.nlm.nih.gov/geo/query/acc.cgi?acc=GSE48946) Several studies have shown that Reg family proteins highly express in experimental pancreatitis and remain steady during the recovery phase^5–9^. The specific knockout of *Reg3b* or administration of anti-Reg1, anti-Reg3a, and anti-Reg4 antibodies worsen experimental pancreatitis, while administration of recombinant Reg3a and Reg4 can significantly reduce the pancreatic damage^10–13^. However, *Reg2* gene deficiency or acinar cell-specific overexpression of *Reg2* does not affect the severity or offer any protection against experimental pancreatitis^7,11^. Additionally, Reg1 has been identified as a novel pancreatic stellate cells (PSCs) activator in the regenerating pancreas^14^. Accordingly, current data suggest that Reg family proteins could serve as essential protective factors for recovery from pancreatic damage.

Despite evidence explaining the role of each Reg family protein, no studies have investigated the potential synergies among this superfamily following pancreas injury. Based on the above information, we hypothesize that Reg family proteins are essential protective regulators following pancreas injury and contribute to organ recovery and resolution of fibrosis. To evaluate this hypothesis, we used the CRISPR/Cas9 system to generate *Reg1-3* (*Reg1,2,3a,3b,3d,3g*) genes global knockout mice and investigated the effect of Reg family protein deficiency in a genetic model of CP. Our present results suggest that Reg family proteins may play a significant role in regulating the expression of proinflammatory cytokines and PSCs activation in CP, which may contribute to pancreatic fibrosis. These findings also indicate that targeting Reg family proteins may be a promising avenue for developing new treatments for CP.

## Results

### Effects of *Reg1-3* genes deficiency on pancreatic development

**Figure 1:**
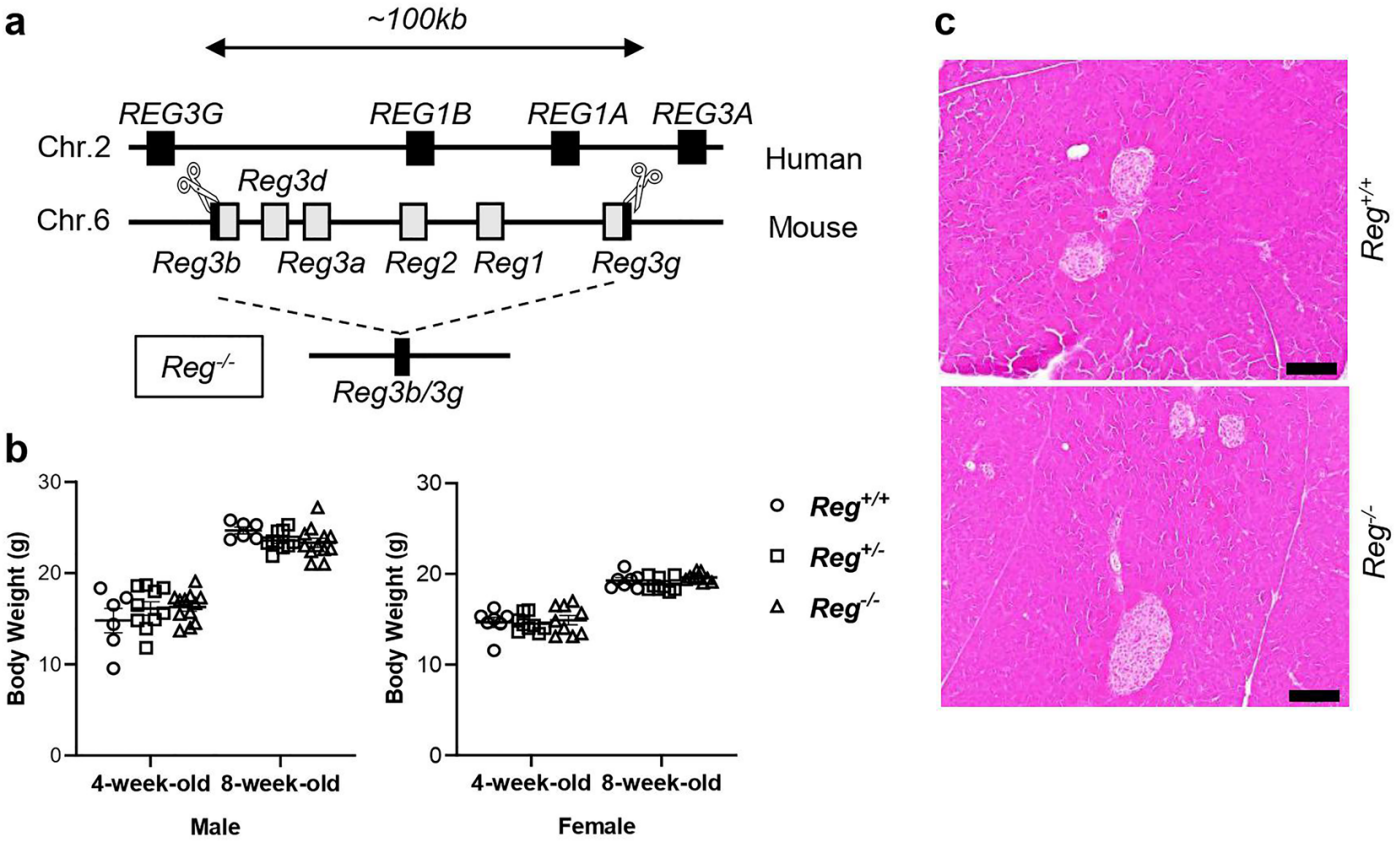
*Reg1-3* genes were not necessary for pancreatic development and general growth in mice. (**a**) Schema of *Reg1-3* genes location of human and mouse. We deleted about 100 kb, including no other genes. (**b**) 4-week-old and 8-week-old body weight among *Reg*^+*/*+^, *Reg*^+*/−*^ and *Reg*^*−/−*^ male and female mice. Bars indicate mean ± SEM (n = 6–12 mice). Statistical analysis was performed by one-way ANOVA with Tukey’s multiple comparison tests. (**c**) Representative hematoxylin and eosin (H&E) staining of pancreas tissue sections isolated from *Reg*^+*/*+^ and *Reg*^*−/−*^ mice at 8-week-old during normal feeding (n = 4 mice). Scale bars: 100 μm.

The *Reg* family in mice contains seven members, with *Reg1-3* located on the same chromosome 6C3 and *Reg4* on 3F3. Mice homozygous for the disrupted *Reg1-3* genes (Fig. 1a) were born at the expected Mendelian ratio, did not exhibit any abnormal growth (Fig. 1b), and were phenotypically indistinguishable from their wild-type or heterozygous littermates. No embryonic lethality or significant developmental defects were observed in the *Reg*^*−/−*^ mice and both male and female adult *Reg*^*−/−*^ mice were fertile and had normal litter sizes. Additionally, microscopic examination of pancreatic tissue under normal feeding conditions showed no morphological differences between *Reg*^+*/*+^ and *Reg*^*−/−*^ mice (Fig. 1c). These findings indicate that the *Reg1-3* genes are not necessary for general growth in mice.

### *Reg* deficiency alleviated pancreatic damage and fibrosis in CP

**Figure 2:**
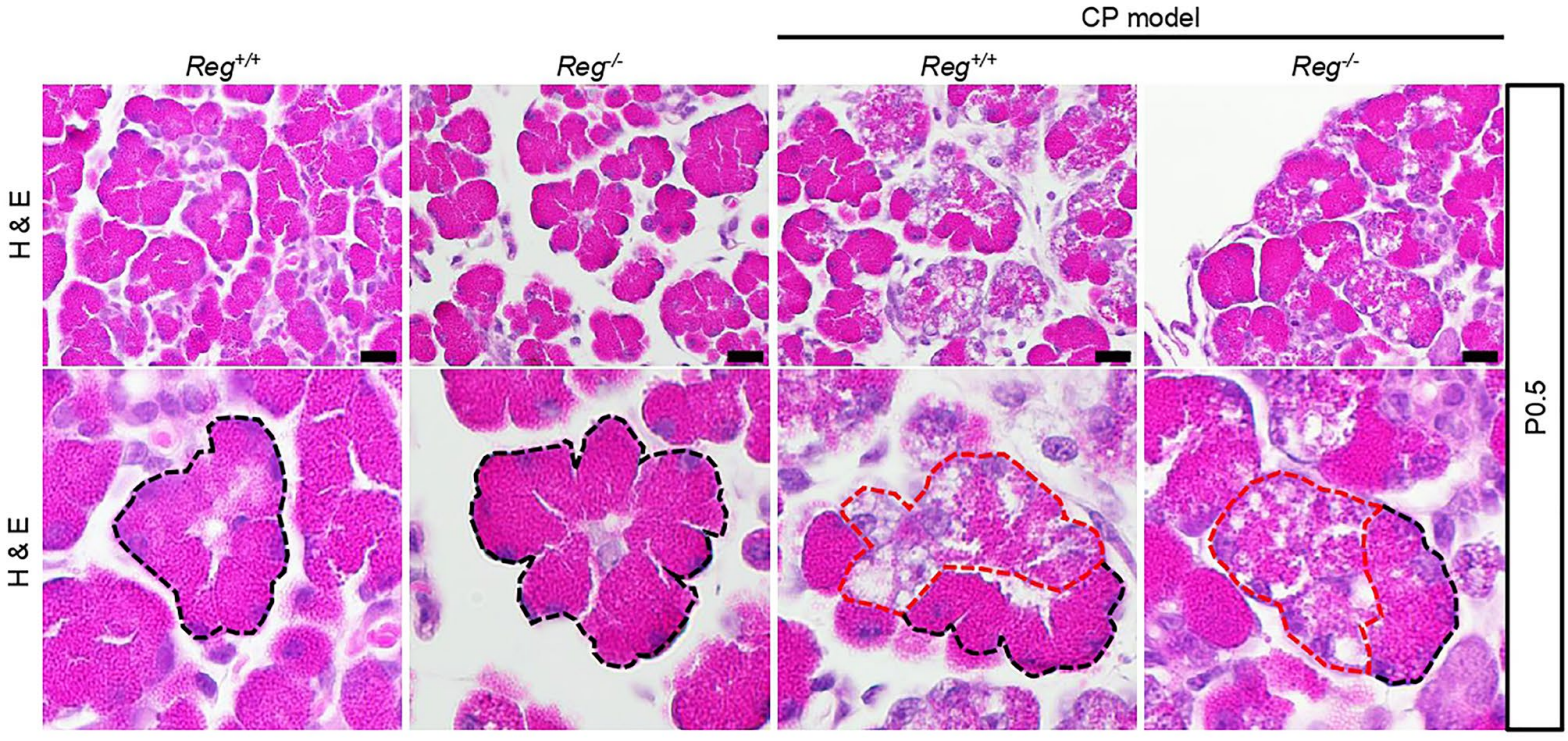
Representative H&E staining of pancreas tissue sections of indicated mice at 0.5 day after birth (P0.5) (n = 3–4 mice). The lower panel show enlarged images of the upper panels. Normal acinar cells were delineated by black dashed lines and those that exhibited vacuolization were by red dashed lines. Scale bars: 20 μm.

**Figure 3:**
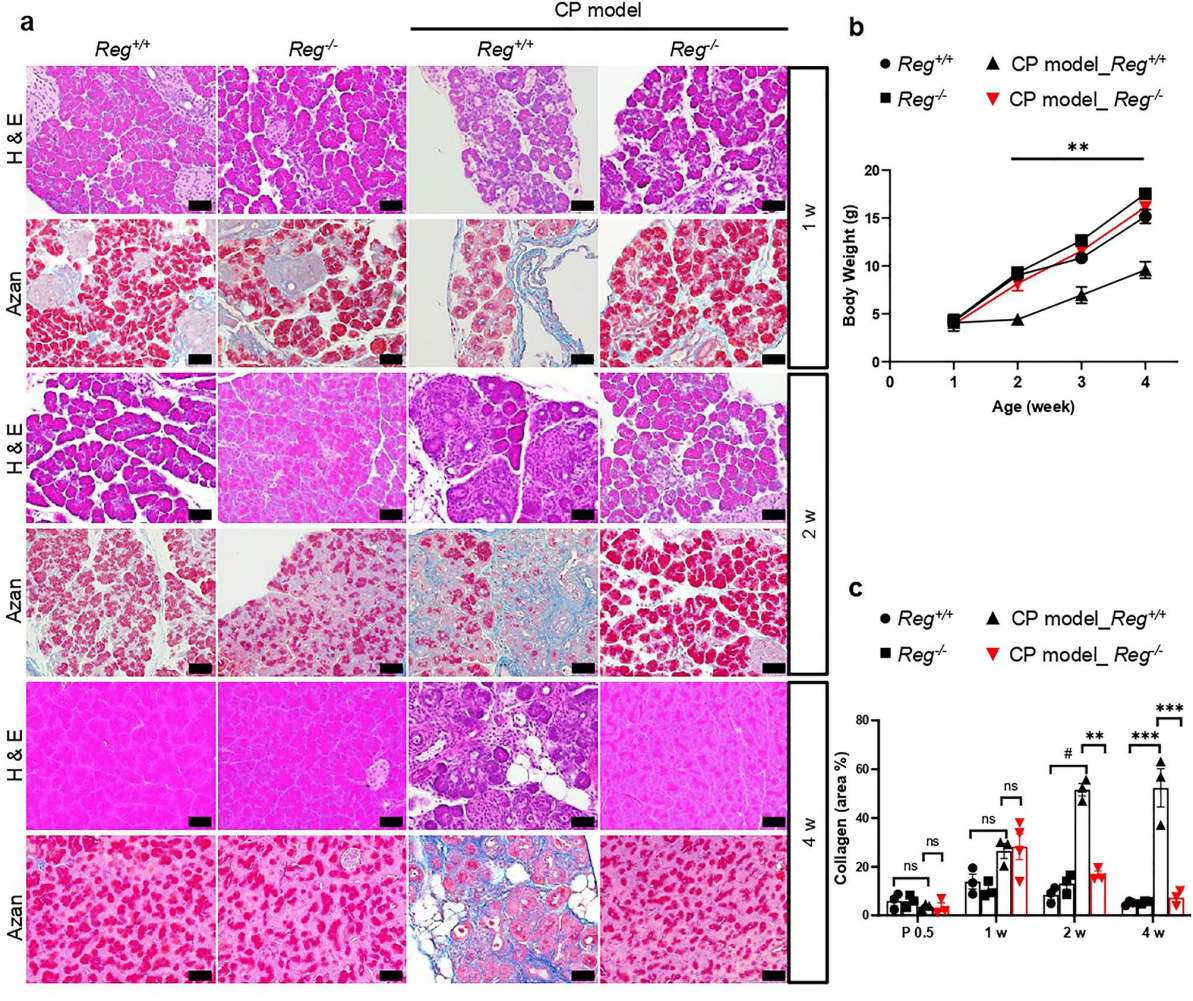
*Reg* deficiency alleviated pancreatic damage and fibrosis in chronic pancreatitis. (**a**) Representative H&E and Azan staining of pancreas sections of indicated mice at 1 week (1w), 2w, and 4w (n = 3–4 mice). Scale bars: 50 μm. (**b**) Body weight gain of the indicated mice. Results represent mean ± SEM (n = 3–7 mice). Statistical analysis was performed by one-way ANOVA among four groups. **P < 0.01 (**c**) The percentage of AZAN-positive staining (for collagen, dark blue). Results represent mean ± SEM (n = 3–5 mice). Statistical analysis was performed by one-way ANOVA with Tukey’s multiple comparison tests among four groups. ns: not significant; **P < 0.01; ***P < 0.001; ^#^*P* < 0.0001.

The human serine protease inhibitor, Kazal type 1 (*SPINK1*), plays a critical role in inhibiting intrapancreatic trypsinogen activation and is considered a key mechanism in preventing the development of pancreatitis. In a previous study, we reported the loss of function of its murine homolog, *Spink3,* resulting in *Spink3*^*−/−*^ mice dying within two weeks after birth^15^. However, we developed a novel method to rescue this lethal phenotype by integrating the human *SPINK1* gene on the X chromosome, resulting in *Spink3*^*−/−*^*; XX*^*SPINK1*^ female mice (hereafter CP model mice). These mice showed a mosaic pattern of *SPINK1* expression due to X chromosome random inactivation and developed spontaneous CP^16^. To investigate the role of the *Reg* gene family in CP, we crossed *Reg*^*−/−*^ mice with CP model mice. As we previously reported, the littermates of CP model mice were smaller with lower body weight, and the acinar cells showed partly vacuolization and partly normal at 0.5 days after birth (P0.5), but the vacuoles were eliminated at 1 week (1 w) after birth (Figs. 2, 3a). These mice gradually developed CP, characterized by loss of acinar cells, inflammatory cells infiltration, fat replacement, and intralobular fibrosis (Fig. 3a). However, although *Reg* deficient mice exhibited similar morphological changes in CP model mice at P0.5 and 1w, the littermates lacking *Reg* genes in CP model grew normally and could not be distinguished from control mice phenotypically. Furthermore, these mice alleviated pancreatic damage and fibrosis characterized by deposition of collagen earlier at 2 weeks of age completely (Figs. 2, 3, Supplementary Fig. 1).

### *Reg* deficiency reduced proinflammatory cytokines expression in CP

**Figure 4:**
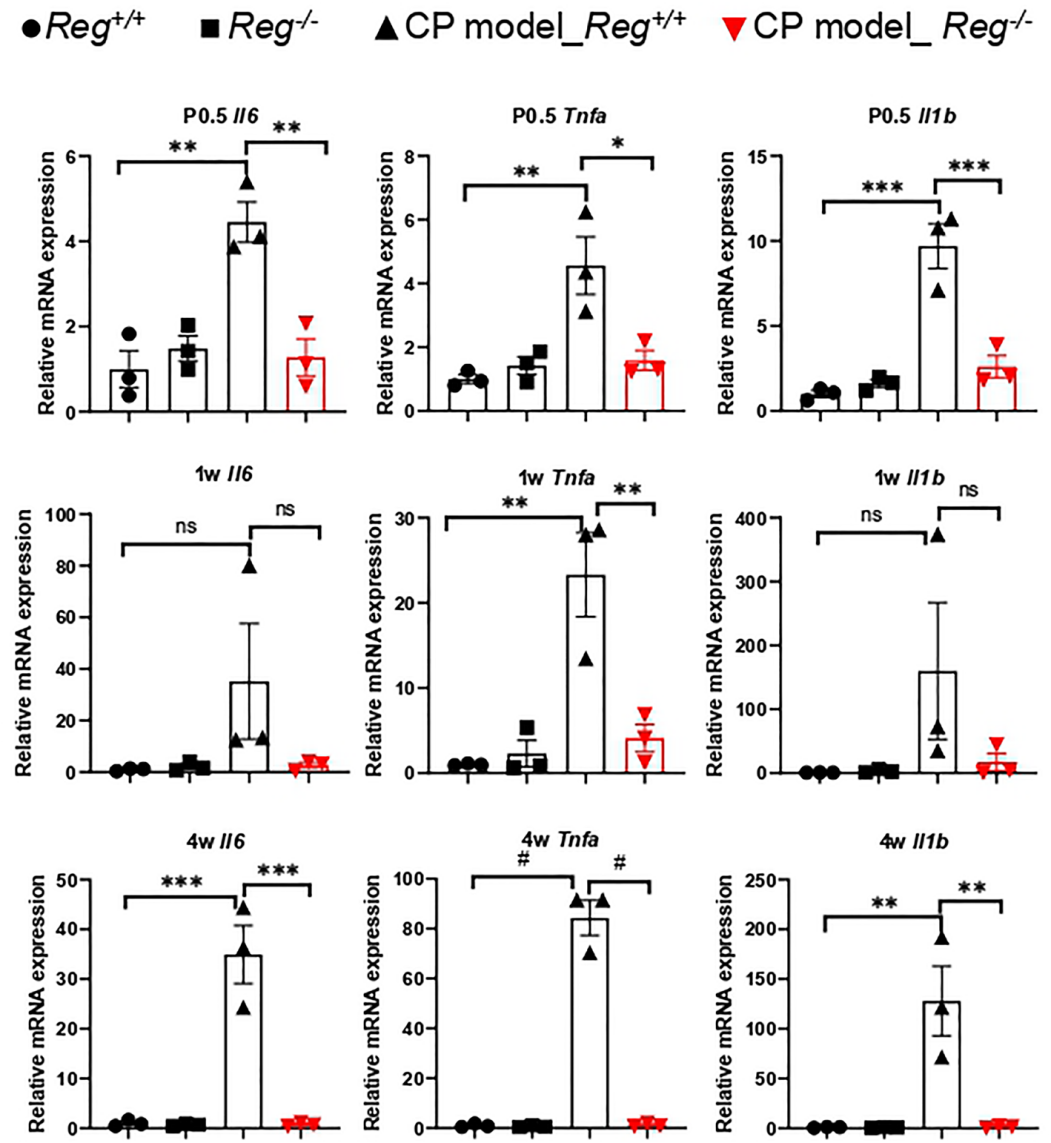
Pancreatic mRNA expression of proinflammatory cytokines interleukin 6(*Il6*), tumor necrosis factor a (*Tnfa*), and *Il1b* of indicated mice at 0.5 day after birth (P0.5), 1 week (1w), and 4w were assessed by qRT-PCR. The expression amounts of each gene were calculated relative to those of ribosomal protein S3 (*Rps3*) with the fold change to the *Reg*^+*/*+^ mice. Mean ± SEM (n = 3 mice). Statistical analysis was performed by one-way ANOVA with Tukey’s multiple comparison tests among four groups. ns: not significant; *P < 0.05; **P < 0.01; ***P < 0.001; ^#^*P* < 0.0001.

Persistent inflammation in the pancreas is considered the underlying mechanism of CP. To investigate the effect of *Reg* deficiency on inflammation, we evaluated the mRNA expression levels of proinflammatory cytokines, including interleukin 6 (*Il6*), tumor necrosis factor alpha (*Tnfa*), and *Il1b*, in CP mice. The expression of these cytokines increased with the progression of CP, but *Reg*^*−/−*^ mice showed significantly reduced levels of these cytokines as early as P0.5, and these reduced levels persisted for up to 4 weeks, similar to control mice (Fig. 4). Distinct local immune cells population were reported in different CP subtypes^17,18^.We did not observe obvious immune cell infiltration at P0.5 in either our hematoxylin–eosin stained or CD154 antibody^19,20^ stained pancreatic sections of all genotypes of mice. After 2 weeks of age, immune cell infiltration (mainly lymphocytes) could be seen in *Reg* sufficient-CP model mice. However, *Reg* deficient-CP model mice did not show evidence of immune cell infiltration during CP (Figs. 2, 3a, Supplementary Fig. 2).These findings suggest that Reg family proteins may play a significant role in regulating immune response and the expression of proinflammatory cytokines in CP.

### Reg proteins stimulated PSCs activation contributing to fibrosis in CP

**Figure 5:**
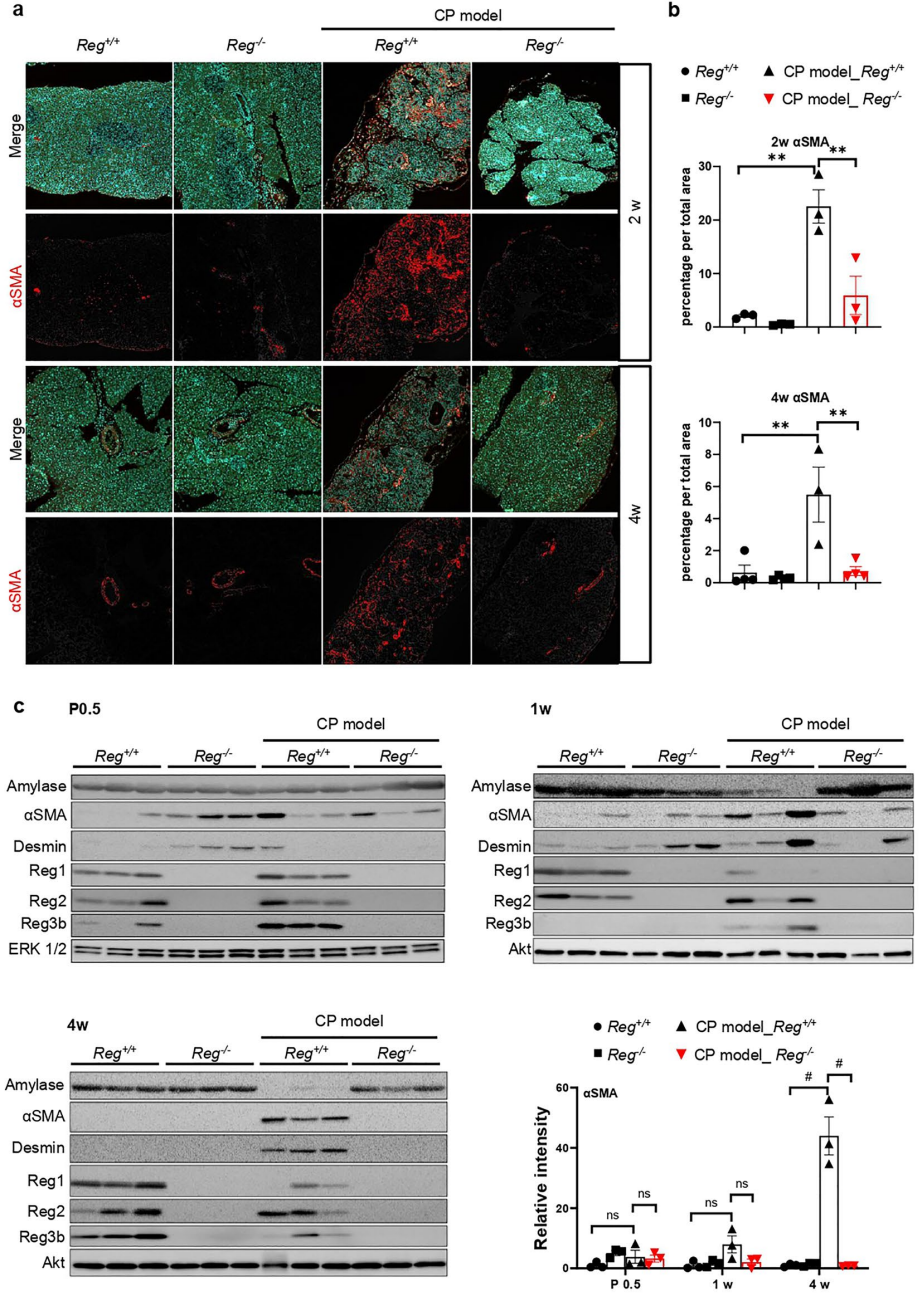
Reg family proteins stimulated PSCs activation contributing to fibrosis in chronic pancreatitis. (**a**) Immunofluorescence analysis of alpha-smooth muscle actin (αSMA, an activated PSCs marker, red) in the pancreas sections of indicated genotypes at 2 weeks, and 4 weeks (n = 3–4 mice). Nuclei were counterstained by DAPI (blue). (**b**) The percentage of epithelial cells with αSMA positive signals. Results represent mean ± SEM (n = 3–4 mice). Statistical analysis was performed by one-way ANOVA with Tukey’s multiple comparison tests among four groups. **P < 0.01 (**c**) Cropped images of western blot gels of Amylase, αSMA, Desmin, Reg1, Reg2, Reg3b of the pancreas of indicated genotypes at 0.5 day after birth(P0.5), 1w and 4w, p44/42 MAPK (ERK 1/2) and Akt were used as the loading control (n = 3 mice). The samples derived from the same experiment and gels were processed in parallel. Images of the entire gels are presented in Supplementary Fig. 5–7. The right panel showed the respective densitometric quantification analysis of the relative intensity of αSMA. Densitometric quantification analysis of other proteins is presented in Supplementary Fig. 4.Results represent mean ± SEM (n = 3 mice). Statistical analysis was performed by one-way ANOVA with Tukey’s multiple comparison tests among four groups. ns: not significant; ^#^*P* < 0.0001.

PSCs are considered to be the primary extracellular matrix-producing cells in the pancreas that activate in experimental models and contribute to organ repair. Previous studies have confirmed Reg1 as a PSCs activator^14^. To determine whether *Reg1-3* deficiency could modify the extent of PSCs activation during CP development, we performed immunofluorescence staining and western blot analysis using the cytoskeletal protein alpha-smooth muscle actin (αSMA), an activated PSCs marker, and Desmin, a quiescent PSCs marker^21^, to monitor and measure the activation of PSCs, pancreatic exocrine function, and Reg protein expression. Consistent with the previous AZAN data, PSCs activation levels were low in all genotypes until 1w and gradually increased significantly during CP development in *Reg* sufficient-CP model mice compared to control and *Reg* deficient-CP model mice (Fig. 5a,b, Supplementary Fig. 3). Western blot analysis confirmed this result (Fig. 5c). As for the pancreatic exocrine function, *Reg* sufficient-CP model mice showed pancreatic exocrine insufficiency indicated by lower pancreatic amylase expression while *Reg* deletion reversed it. And as per our previous microarray analysis, Reg protein expression increased at P0.5 of CP model mice but appeared to decrease during the development of CP, and indeed, they did not express in *Reg*^*−/−*^ mice as expected (Fig. 5c, Supplementary Fig. 4). These results indicate that Reg protein plays a significant role in PSCs activation, which mediates the development of pancreatic fibrosis in CP.

**Figure 6:**
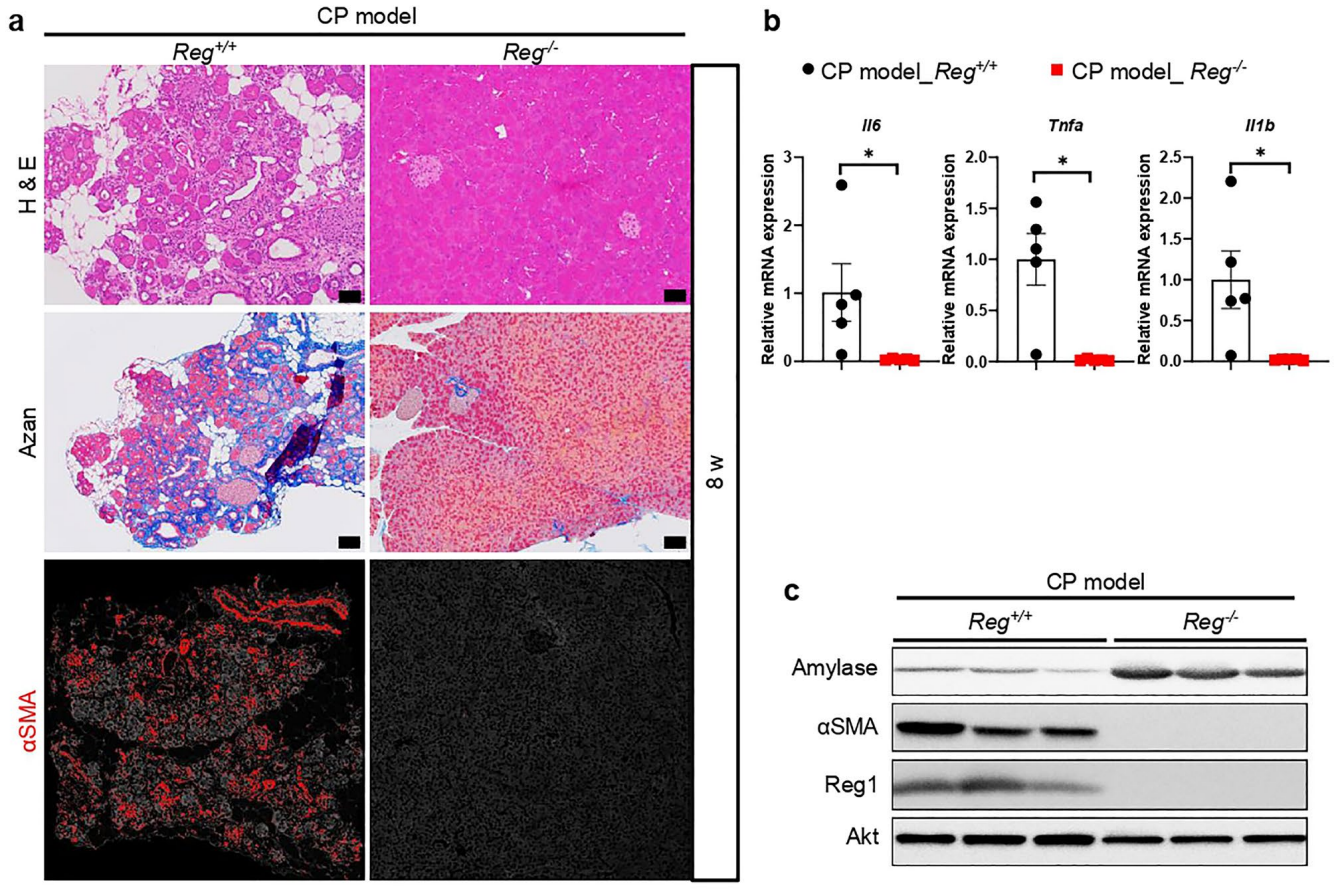
*Reg* deficiency led to the remission of chronic pancreatitis. (**a**) Representative pancreatic tissue section from mice of the indicated genotype at 8 weeks was stained with H&E or Azan (for collagen, dark blue) and Immunofluorescence staining of alpha-smooth muscle actin (αSMA, an activated PSCs marker, red) (n = 5 mice). Scale bars: H&E, 50 μm; Azan, 100 μm (**b**) Pancreatic mRNA expression of proinflammatory cytokines interleukin 6(*Il6*), tumor necrosis factor a (*Tnfa*) and *Il1b* of indicated genotypes were assessed by qRT-PCR. The expression amounts of each gene were calculated relative to those of ribosomal protein S3 (*Rps3*) with the fold change to CP model*_Reg*^+*/*+^ mice. Mean ± SEM (n = 5 mice). Statistical analysis was performed by two-tailed unpaired Student t-test between two groups. *P < 0.05 (**c**) Cropped images of western blot gels of Amylase, αSMA and Reg1 of the pancreas of indicated genotypes at 8 weeks and Akt was used as the loading control (n = 5 mice). The samples derived from the same experiment and gels were processed in parallel. Images of the entire gels and respective densitometric quantification analysis of the relative intensity of protein are presented in Supplementary Fig. 4,8.

We further confirmed *Reg* knockout alleviated pancreatic damage, fibrosis, and the inflammatory response in CP at 8 weeks (Fig. 6a–c, Supplementary Fig. 4). Therefore, our study suggests that *Reg* deficiency may have therapeutic potential for CP by reducing proinflammatory cytokines expression and PSCs activation.

## Discussion

All members of the *Reg* family are expressed in the pancreas^22–24^ and most of them express primarily in pancreatic exocrine and are linked to pancreatitis. By utilizing a novel genetic model of *Reg* to investigate the role of Reg family proteins in the development of CP in mice, the present study has demonstrated that the absence of Reg family proteins reduces the expression of proinflammatory cytokines and alleviates pancreatic damage, fibrosis, and inflammatory response in CP. These results suggest that targeting Reg family proteins could be a potential therapeutic strategy for treating CP.

The reduction in proinflammatory cytokine expression in *Reg*^*−/−*^ mice is a promising result, as these cytokines are known to play a central role in the inflammatory response that underlies the development of CP. Interestingly, our result revealed a surprising finding of an early peak in proinflammatory cytokines mRNA expression at P0.5 in *Reg*-sufficient CP model mice. Intriguingly, this rise in cytokines expression occurred despite the absence of inflammatory cell infiltration or PSCs activation, as observed in Fig. 2, Supplementary Fig. 2 and Supplementary Fig. 3. We propose that this early peak in cytokines expression is a response induced by Reg proteins since the *Reg*-deficient CP model mice group showed comparable levels to the control group. Additionally, we speculate that the induction of proinflammatory cytokines may also stimulate the expression of Reg family proteins from acinar cells. As CP progresses, Reg family proteins could attract inflammatory cells and activate PSCs, which might serve as additional sources of elevated proinflammatory cytokines. Furthermore, the activated PSCs, in turn, could perpetuate inflammation by activating more PSCs. These findings suggest complex interactions among Reg family proteins, proinflammatory cytokines, immune cells, and PSCs, leading to sustained inflammation and fibrosis in CP. The exact regulatory roles of Reg family proteins in immune response need to be further investigated.

PSCs are a multifunctional cell type found in the areas of the pancreas^25^. Activated PSCs have been found in several animal models of experimental pancreatitis facilitating tissue repair or promoting fibrosis^26–28^. Furthermore, Reg1 could inhibit the activity of activated PSCs to have a protective role in the repair phase of damage and was identified as an activator of PSCs following cerulein-induced pancreatitis^3,14^. In contrast to our hypothesis, our present study indicates that *Reg* deficiency contributes to organ recovery and the resolution of fibrosis in CP. This finding differs from the general protective roles suggested by previous studies using single *Reg* gene knockout mice or the administration of recombinant Reg antibodies in different AP models. One possible explanation is that *Reg* functions differently in AP and CP. In AP, the expression of Reg family proteins is upregulated and sustained during the recovery phase, with transient activation of PSCs promoting tissue repair. However, in CP, Reg family proteins increase only at the beginning of pancreatic damage and then decreased as acinar cell loss occurs, with persistent PSCs activation promoting fibrosis. Another explanation could be the integrated function of the *Reg* gene family as we knock out all *Reg1-3* genes. The reduction in PSCs activation observed in *Reg*-deficient mice in this study suggests that targeting Reg family proteins could be a promising approach for preventing or reducing the development of pancreatic fibrosis in CP.

However, it should be noted that this study only focused on the effects of Reg family protein deficiency in a genetic model of CP, and it is unclear whether these findings can be generalized to other models, human patients with CP, or other fibrosis-related diseases. Moreover, the CRISPR/Cas9 system was used to generate global knockout mice for *Reg1-3*, which may not accurately reflect the partial or temporal changes in Reg protein expression that occur in CP. Additionally, the endocrine function was not investigated in this CP model, despite the possibility that *Reg* deficiency could result in injury to the islet β cells in CP^29^. Since we knockout *Reg1-3* genes before the onset of CP, investigating the role of Reg family proteins in the pancreas after the onset of chronic inflammation is of great interest. Addressing these limitations and conducting further research will be crucial in advancing our understanding of the potential roles of Reg family proteins in CP and determining the clinical relevance of these findings.

Overall, this study provides new insights into the role of Reg family proteins in the development of CP and suggests that they could be a promising therapeutic target for treating this disease. Further studies should investigate the potential for targeting Reg proteins to develop new treatments for CP. In this regard, we have generated *Reg1-3* genes floxed mice, and ongoing work in our laboratory aims to explore these aspects.

## Materials and methods

### Antibodies and reagents

Antibodies used in this study were against Amylase (sc-12821, Santa Cruz), alpha smooth muscle Actin (αSMA, ab5694, abcam), Desmin (ab32362, abcam), Reg1 (AF1657, R&D), Reg2(AF1658, R&D), Reg3b(AF1660, R&D), Akt (#9272, CST),p44/42 MAPK (Erk1/2, #9102, CST) ; Horseradish peroxidase-conjugated donkey anti-rabbit IgG (NA9340, GE Healthcare Life Sciences), rabbit anti-goat IgG(AP106P, CHEMICON), rabbit-anti-sheep IgG (#313-035-003, Jackson Immuno Research Laboratories).

### Animal use

Mice were kept under specific-pathogen-free conditions with ad libitum access to food and water in a 12 h light/dark cycle. C57BL/6N mice were purchased from CREA Japan. All animal experiments were approved by the Institutional Animal Care and Use Committee of Hyogo Medical University and performed according to the guidelines approved by the Institutional Animal Care and Use Committee of Hyogo Medical University. The study is reported in accordance with ARRIVE guidelines.

### Generation of ***Reg***^***−/−***^ mice by the CRISPR-Cas9 method

#### Preparation of the Cas9/gRNA complex for generation of *Reg* deficient mice

CRISPR/Cas-mediated genome engineering was conducted to create *Reg* deficient mice. To target intron1 of the mouse *Reg3b* and *Reg3g* genes, gRNA for both murine *Reg3b* and *Reg3g* were designed using CRISPR direct (https://crispr.dbcls.jp). The sequences of the guide for target DNA were as follows: *Reg3b* -G (5′-CTG TCT TTC TCC TGT GAT AC-3’) and *Reg3g* -G (5′-AAG AAG GGG AGA ATT AGT GT-3′), which were located at intron1 of both *Reg3b* and *Reg3g*, respectively. The corresponding crRNA, tracrRNA, and Cas9 nuclease were purchased from Integrated DNA Technologies (IDT). The crRNA and the tracrRNA were annealed and complexed with Cas9 nuclease according to the manufacturer’s instructions.

#### In vitro fertilization (IVF)

We used C57BL/6N female mice (purchased from CREA Japan Inc., Tokyo, Japan) in this study. IVF was performed according to the Center for Animal Resources and Development’s (at Kumamoto University, Japan) protocol (http://card.medic.kumamoto-u.ac.jp/card/english/sigen/manual/onlinemanual.html).

Electroporated embryos were cultured in KSOM medium (ARK Resource, Kumamoto, Japan), and transferred the next day to the oviducts of pseudo-pregnant Jcl: ICR female mice (CLEA Japan).

#### Electroporation

NEPA21 Super Electroporator (NEPAGENE, Chiba, Japan) and 1 mm gap electrode (CUY501P1-1.5, NEPAGENE, Chiba, Japan) were used for electroporation. Zygotes were washed with Opti-MEM (Thermo Fisher Scientific) and then placed in the electrode gap filled with 5 µl of Opti-MEM solution containing 200 ng/µl Cas9 protein and 6 pmol/µl gRNA (crRNA/tracrRNA complex). The eggs were then cultured in KSOM medium at 37 °C and 5%CO2 in an incubator until the two-cell stage.

#### Genomic DNA extraction, genotyping, and sequencing

Genomic DNA was extracted from F0 mice. PCR was performed using Taq DNA polymerase (Greiner). The primer sequences were as follows: RegKO-S 5’-CCACCATCCTTAACTGGATC-3′; RegKO-AS 5’-CTAGAGTCCATGCCAAGCAC -3′. The PCR products were purified using SV Gel and PCR Clean-Up System (Promega). The purified PCR product were sequenced using BigDye Terminator v3.1 Cycle Sequencing Kit (Thermo Fisher Scientific) through Applied Biosystems 3500xL Genetic Analyzer (Thermo Fisher Scientific).

### Chronic pancreatitis model and tissue processing

In the CP model, X-SPINK1 (*Spink3*^*−/−*^*; XX*^*SPINK1*^) mice were generated and maintained in our animal facility as previously reported^16^. *Reg1-3* genes additionally knockout in *X-SPINK1* mice were obtained by mating male *Reg*^+*/−*^ mice with female X-SPINK1 mice. The resulting offspring were further intercrossed with each other to generate mice used for this study.

Female mice at different ages (P0.5,1w,2w,4w,8w) were immediately sacrificed by cervical dislocation. The pancreas was removed, cut into three pieces, and immediately frozen in liquid nitrogen and store at − 80 °C for subsequent studies, or fixed in 10% neutral buffered formalin for histological analysis.

### Histology and immunofluorescence analysis

Histology and Immunofluorescence analysis were conducted on pancreas tissue. The tissue was fixed in 10% neutral buffered formalin overnight and then paraffin-embedded, sectioned, and stained with hematoxylin–eosin (H&E) or Azan using standard procedures. To quantify the fibrosis score, RGB color images of AZAN staining were taken using a BX-53 microscopy system. The RGB colors were separated into R/G/B single images, and the fibrosis score was calculated as the percentage of Blue-substrate-Red area/total area, using NIH Image J software (http://rsb.info.nih.gov/ij/).

For the Immunofluorescence staining, paraffin-embedded sections were used with the listed antibodies. To quantify the positive staining area, RGB color images of Immunofluorescence staining were taken using a LSM780 microscopy system with ZEN software. The RGB colors were separated into R/G/B single images, and the positive staining area was calculated as the percentage of Red (or Green)-substrate-Blue area/total area, using NIH Image J software (http://rsb.info.nih.gov/ij/).

### RNA extraction and semi-quantitative real-time PCR (qRT-PCR)

Total RNA was extracted from the pancreas using the RNeasy Plus Universal Mini Kit (Qiagen) following the manufacturer's instructions. RNA concentration was measured using a spectrophotometer (Bio Spec-nano, Shimadzu Biotech). Total RNA (2 μg) was reverse-transcribed into cDNA using the ReverTra Ace qPCR RT Master Mix with gDNA Remover (Toyobo) and qRT-PCR assays were carried out using the Thermal Cycler Dice Real-Time PCR System III (Takara) with THUNDERBIRD SYBR qPCR Mix (Toyobo). qRT-PCR was performed with 50 ng of RNA equivalent cDNA and the cycling conditions were as follows: 95 °C for 1 min, followed by 40 cycles of 95 °C for 15 s, 60 °C for 1 min, and 60 °C for 1 min. Amplification specificity was confirmed using the melting curves and gel electrophoresis. The relative amounts of target mRNA were determined by 2^−ΔΔCT^ method relative to those of ribosomal protein S3 (*Rps3*) mRNA expression levels. Values were presented as fold changes against the control group. Details of the qRT-PCR primers are shown in Supplementary Table 1.

### Western blotting

Total proteins of pancreas tissue samples were extracted in ice-cold RIPA buffer (50 mM Tris–HCl, pH 7.2, 150 mM NaCl, 1% Deoxycholic acid,0.1% SDS,1% Triton X-100 with freshly added protease inhibitor cocktail [1:100 dilution; Nacalai Tesque,25,955–11] and PMSF [1:100 dilution; Nacalai Tesque]). The protein concentration was determined by the Pierce BCA Protein Assay Kit (Thermo Scientific) and normalized to same protein amount. The samples were denatured by boiling for 5 min in Laemmli sample buffer containing 10% Dithiothreitol. Equal protein extracts were separated by sodium dodecyl sulfate–polyacrylamide gel electrophoresis and transferred to Immobilon-P polyvinylidene difluoride membranes (Millipore). After blocking with 5% non-fat dry milk in TTBS (0.1% Tween 20 in Tris-buffered saline) for 1 h at room temperature, membranes were incubated overnight at 4 °C with primary antibodies diluted in TTBS containing 5% non-fat dry milk. Membranes were then washed three times with TTBS (10 min each) and finally incubated with horseradish peroxidase conjugated secondary antibody diluted in TTBS containing 5% non-fat dry milk for 1 h at room temperature followed by three washes with TTBS (10 min each). The membranes were developed with Chemi-Lumi one or super (Nacalai Tesque) and analyzed in WSE-6100H LuminoGraph1(Atto). ERK 1/2 and Akt were used as loading controls. Uncropped scans of membranes are shown in Supplementary Fig. 5–8. Densitometric quantification was carried out using Image J software (http://rsb.info.nih.gov/ij/).

### Statistical analysis

Statistical analysis was performed using GraphPad Prism software, version 8.0.2. The normality and lognormality of data were assessed using the Shapiro–Wilk test. The two-tailed unpaired Student t-test was used to determine statistical significance between two groups, and one-way ANOVA was used for comparisons between more than two groups. If normality was not met, the Kruskal–Wallis test was used. Results were considered statistically significant if the p-value was less than 0.05. Data are presented as mean ± standard error of the mean (SEM).

## Supplementary Information

Supplementary Information.

## Data Availability

The datasets analysed during the current study are available in the Gene Expression Omnibus (GEO) repository, https://www.ncbi.nlm.nih.gov/geo/query/acc.cgi?acc=GSE48946.
