# Supplementary material for: Reg family proteins contribute to inflammation and pancreatic stellate cells activation in chronic pancreatitis
Source: Sci Rep. 2023 Jul 27;13:12201. doi: 10.1038/s41598-023-39178-3 (PMC10374637; doi:10.1038/s41598-023-39178-3)
Supplement: Supplementary file 1 — Supplementary Information. [file 41598_2023_39178_MOESM1_ESM.docx]

**Supplementary information for**

**Reg family proteins contribute to inflammation and pancreatic stellate cells activation in chronic pancreatitis**

Wenting Chen, Mai Imasaka, Miyu Lee, Hirokazu Fukui, Hiroshi Nishiura, Masaki Ohmuraya

Corresponding author: Masaki Ohmuraya

Email: ohmuraya@hyo-med.ac.jp

**Supplementary Table 1. Primer Sequences for qRT-PCR**

| Genes | Forward primer (5′-3′) | Reverse primer (5′-3′) |
| --- | --- | --- |
| *Il1b*  *Il6*  *Rps3*  *Tnfa* | AGGTCGCTCAGGGTCACAAG  CCACTTCACAAGTCGGAGGCTTA  ATCAGAGAGTTGACCGCAGTTG  CATCTTCTCAAAATTCGAGTGACAA | GTGCTGCCTAATGTCCCCTTGAATC  GCAAGTGCATCATCGTTGTTCATAC  AATGAACCGAAGCACACCATAG  TGGGAGTAGACAAGGTACAACCC |

**Supplementary Table 1.** Primer Sequences for qRT-PCR. Interleukin (*Il*), tumor necrosis factor (*Tnf*), ribosomal protein S (*Rps*).

**Supplementary Figures and Figure legends**

**
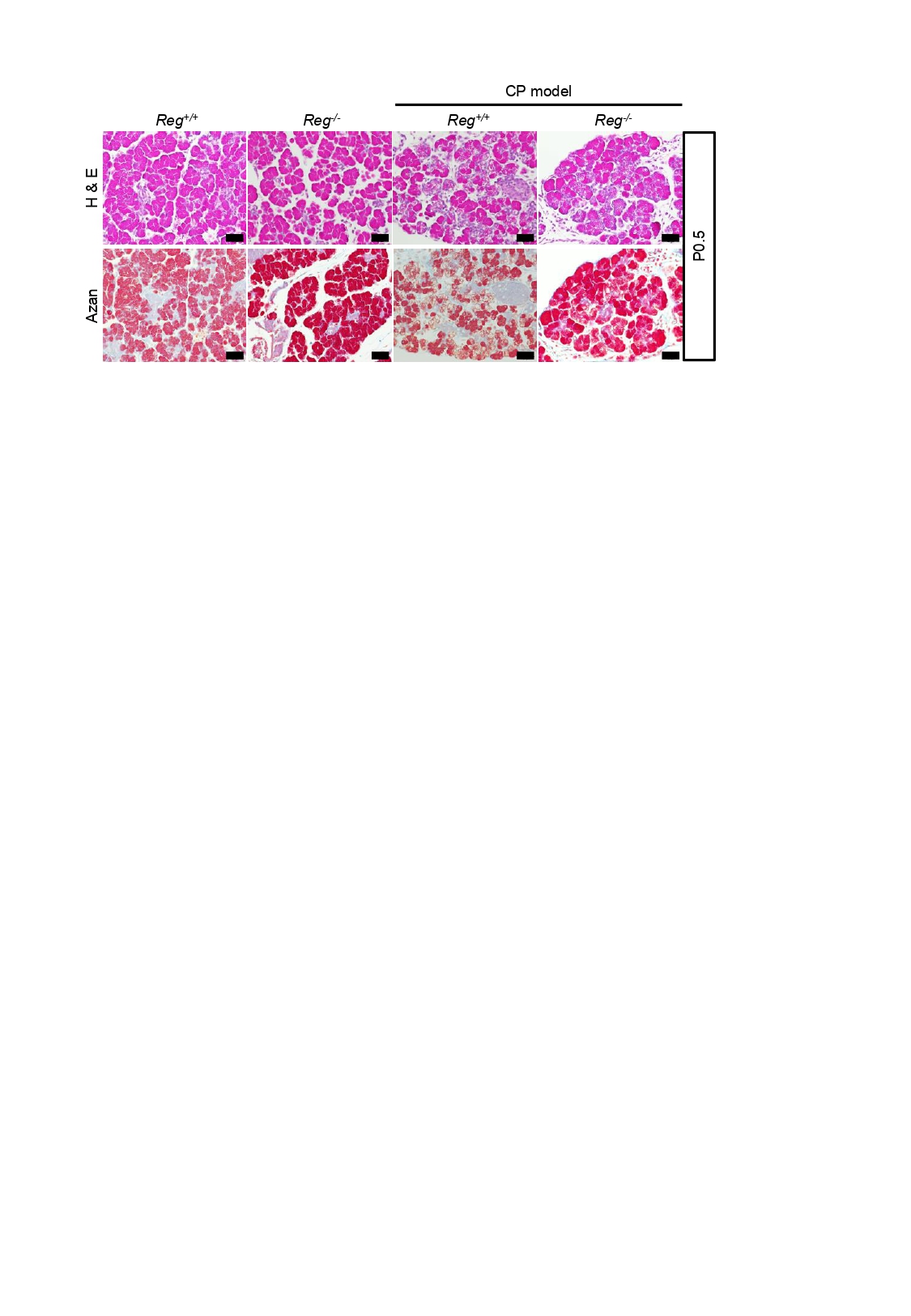
**

**Supplementary Fig.1** Hematoxylin and eosin （H&E）or Azan (for collagen) stained pancreatic tissue sections at 0.5 day after birth（P0.5）from mice of indicated genotype (n=3-4 mice). Scale bar:50μm.


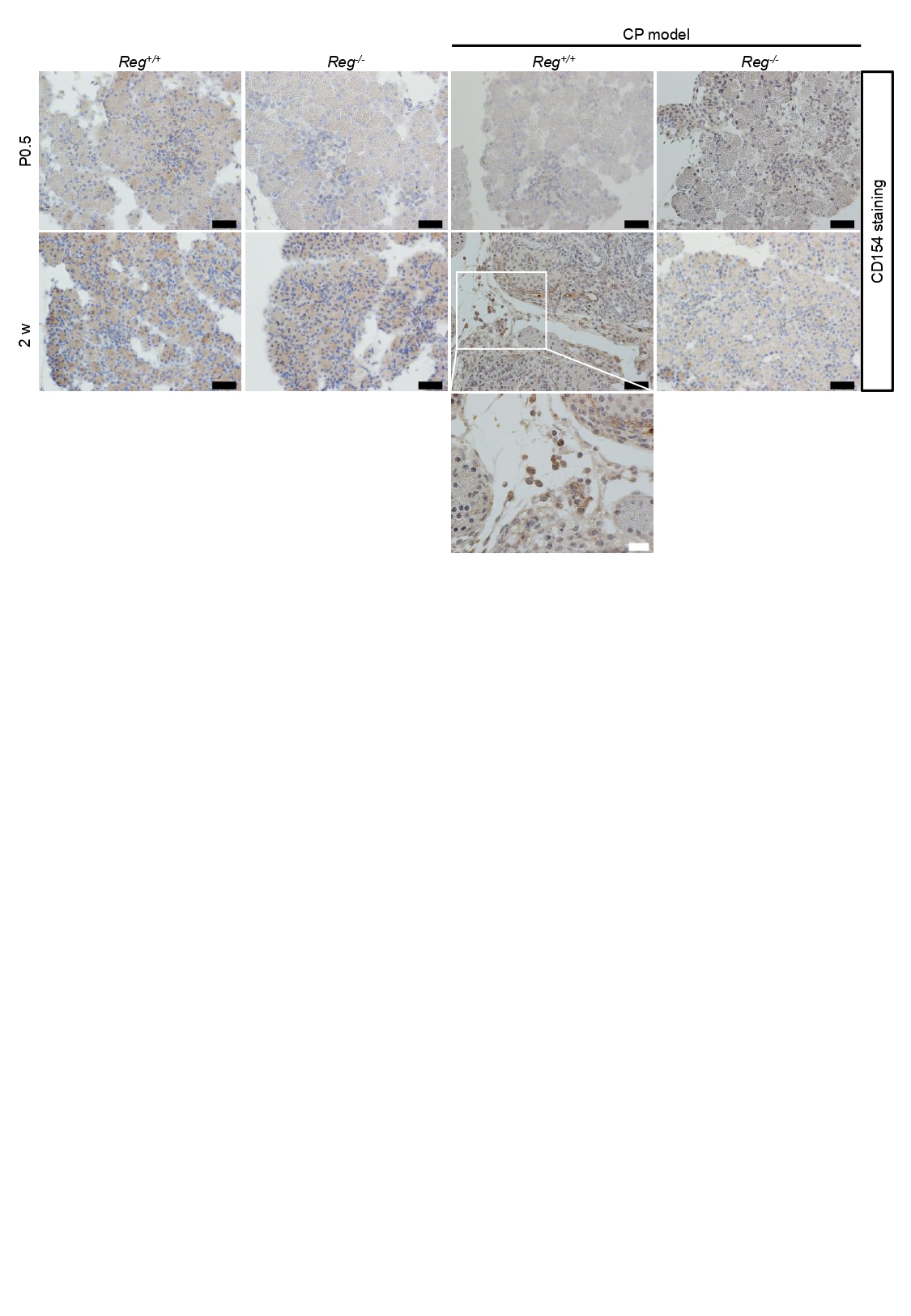


**Supplementary Fig.2** Immunohistochemistry staining of CD154(brown, activated T cells marker) stained pancreatic tissue sections at 0.5 day after birth（P0.5）and 2 weeks from mice of indicated genotype. The lower panel showed a higher magnification picture of the white square area. Scale bar: black 50μm; white 20μm.


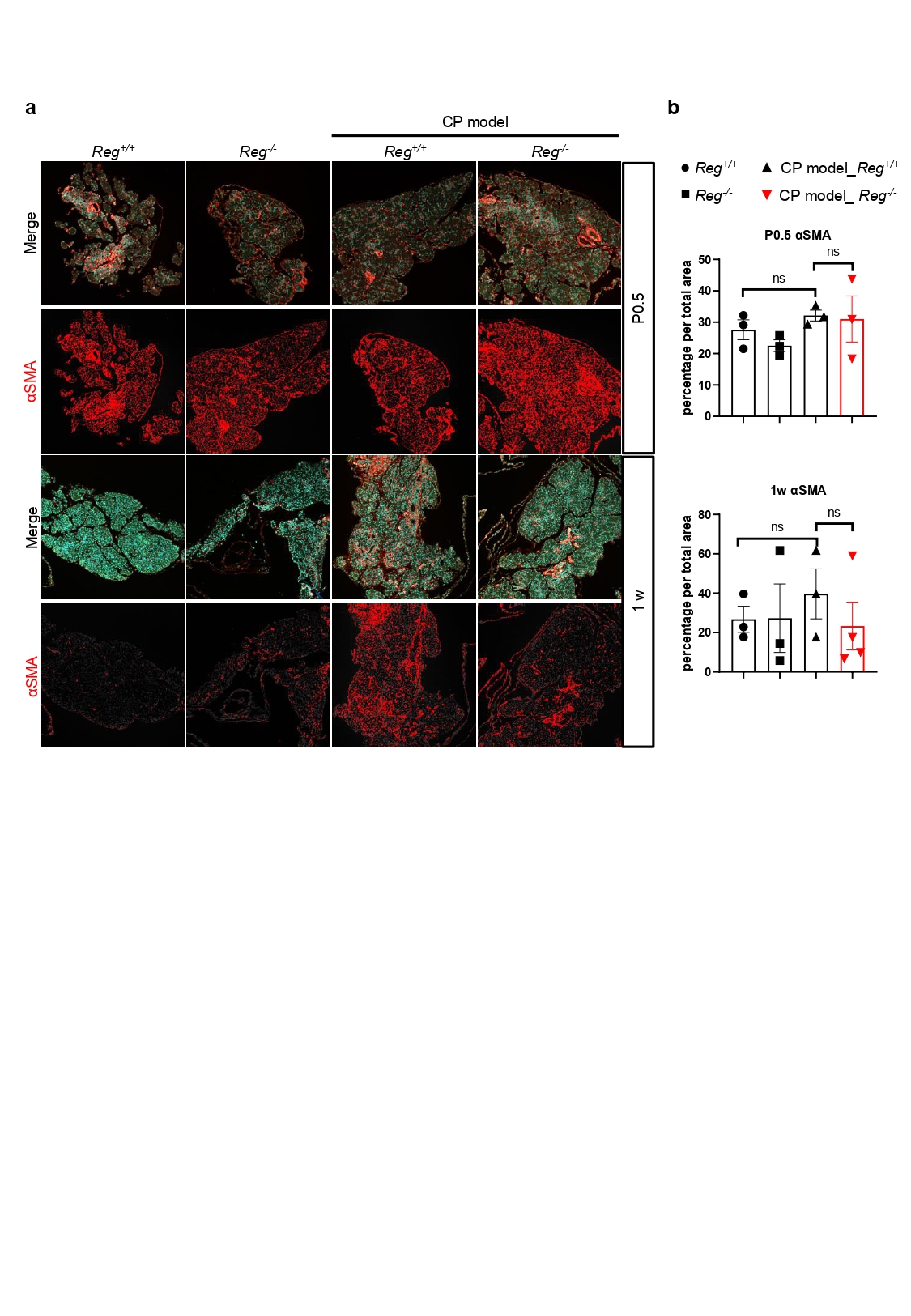


**Supplementary Fig.3** (a)Immunofluorescence analysis of alpha-smooth muscle actin (αSMA, an activated PSCs marker, red) in the pancreas sections of indicated genotypes at 0.5 day after birth (P0.5) and 1 week (n=3-4 mice). Nuclei were counterstained by DAPI (blue). (b)The percentage of epithelial cells with αSMA positive signals. Results represent mean ± SEM (n=3-4 mice). Statistical analysis was performed by one-way ANOVA with Tukey’s multiple comparison tests among four groups. ns: not significant


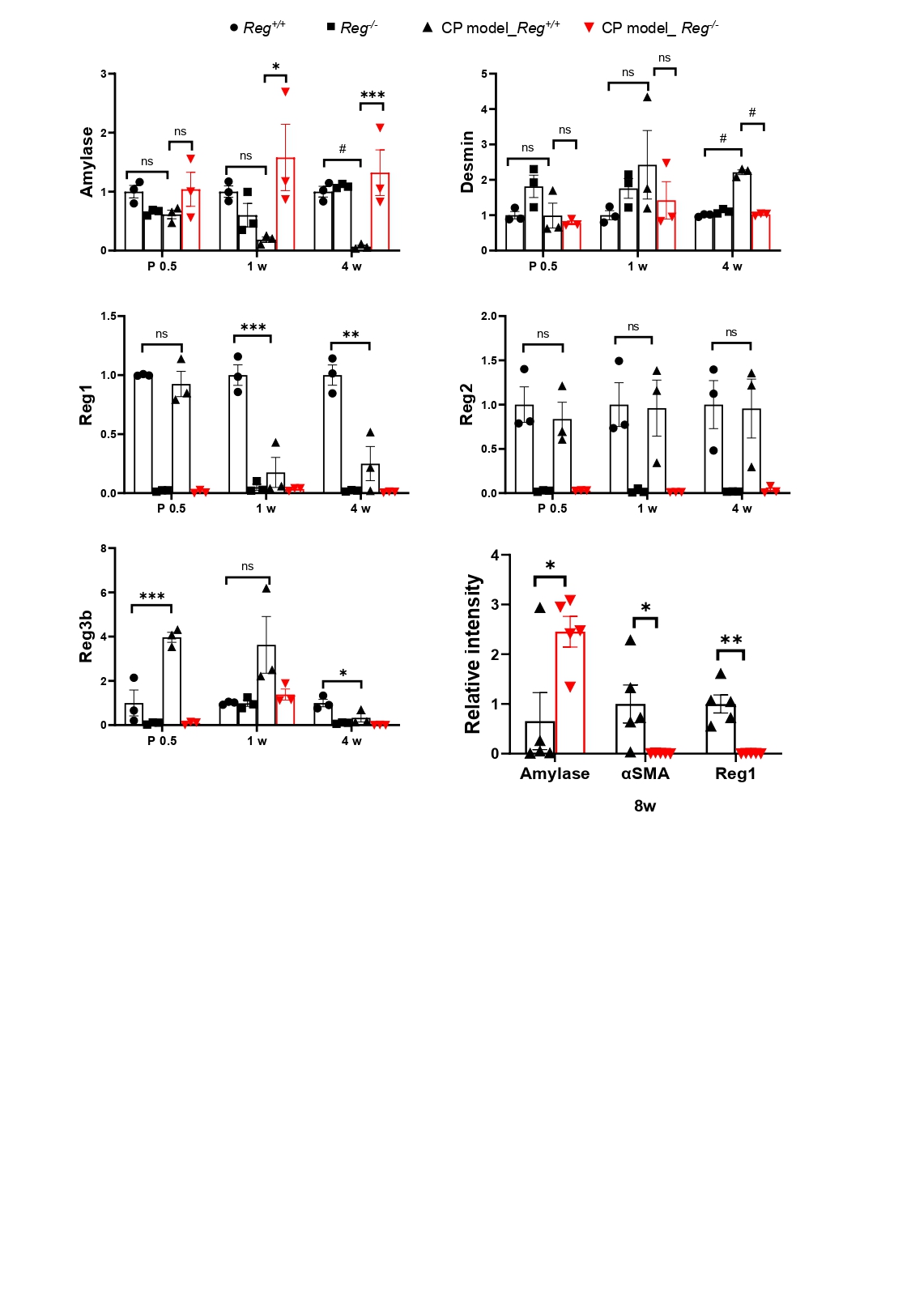


**Supplementary Fig.4** Respective densitometric quantification analysis of the relative intensity of Amylase, Desmin, Reg1, Reg2, Reg3b of Fig.5c and Amylase, αSMA, and Reg1 of Fig.6c. Results represent mean ± SEM（n=3-5 mice). Statistical analysis was performed by one-way ANOVA with Tukey’s multiple comparison tests among four groups or a two-tailed unpaired Student t-test between two groups. ns: not significant; *P<0.05; **P<0.01; ***P<0.001; #P<0.0001


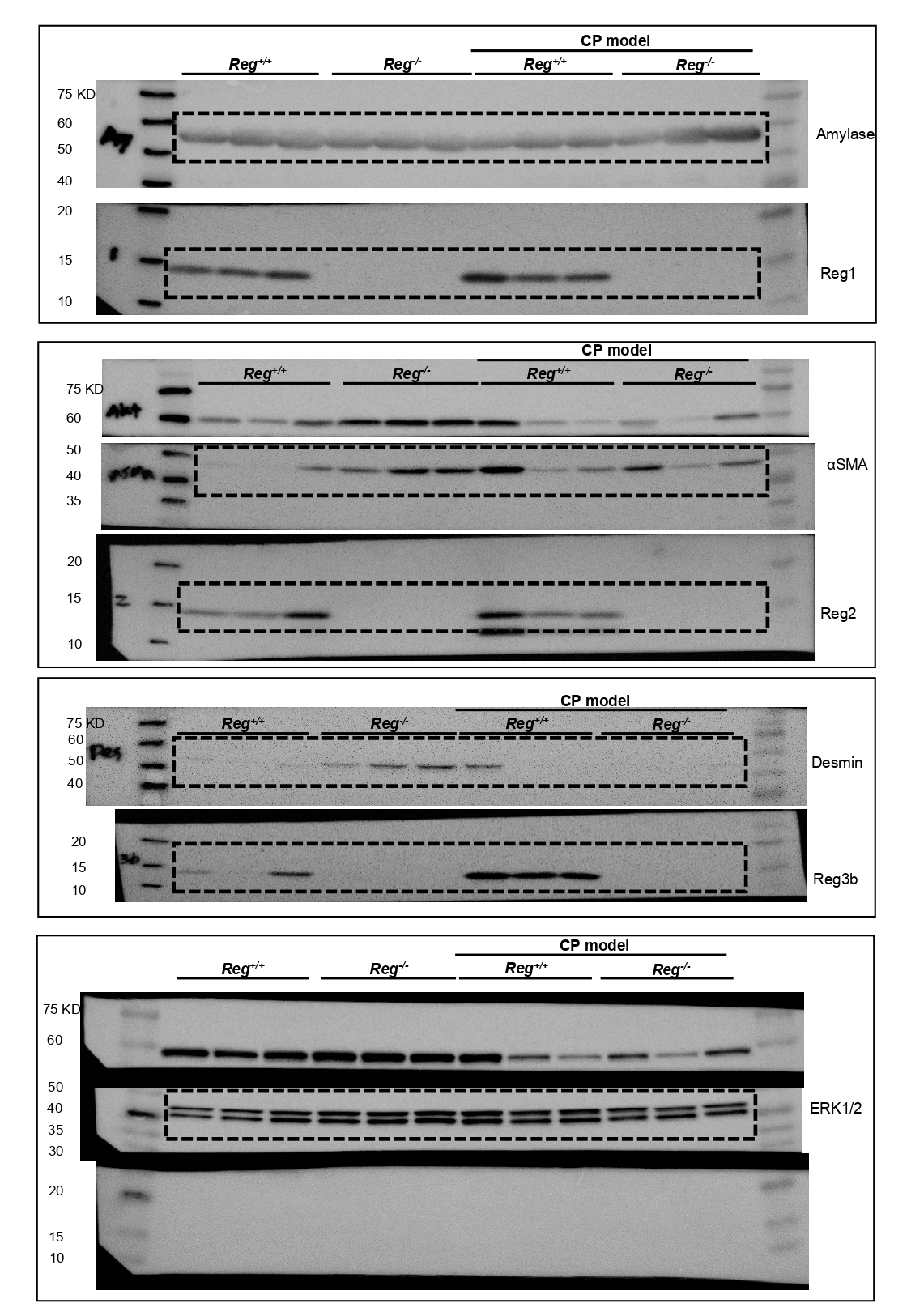


**Supplementary Fig.5** Images of the entire western blots for Fig.5c at 0.5 day after birth (P0.5). Dashed line indicated cropped part of each protein.


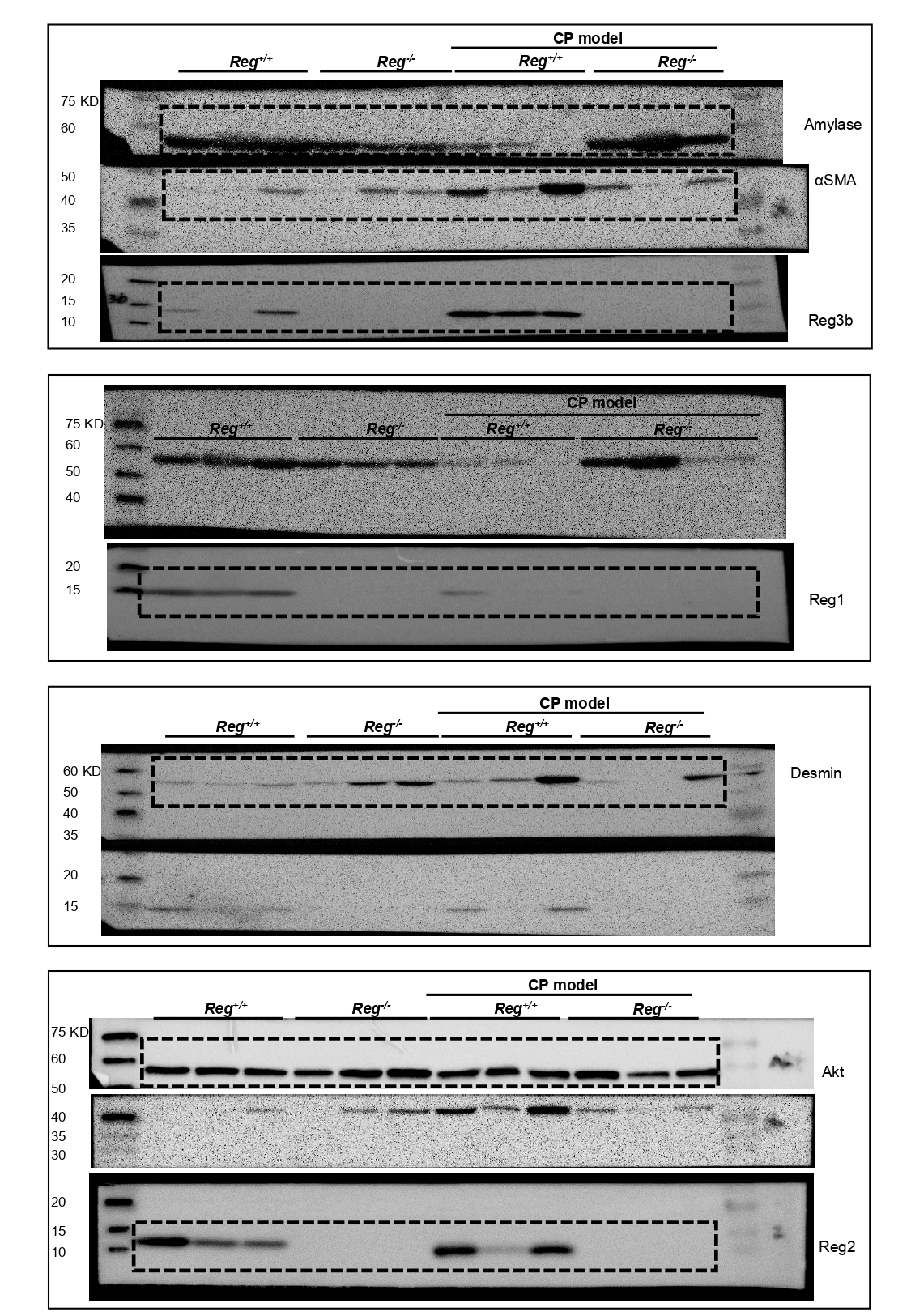


**Supplementary Fig.6** Images of the entire western blots for Fig.5c at 1week. Dashed line indicated cropped part of each protein.


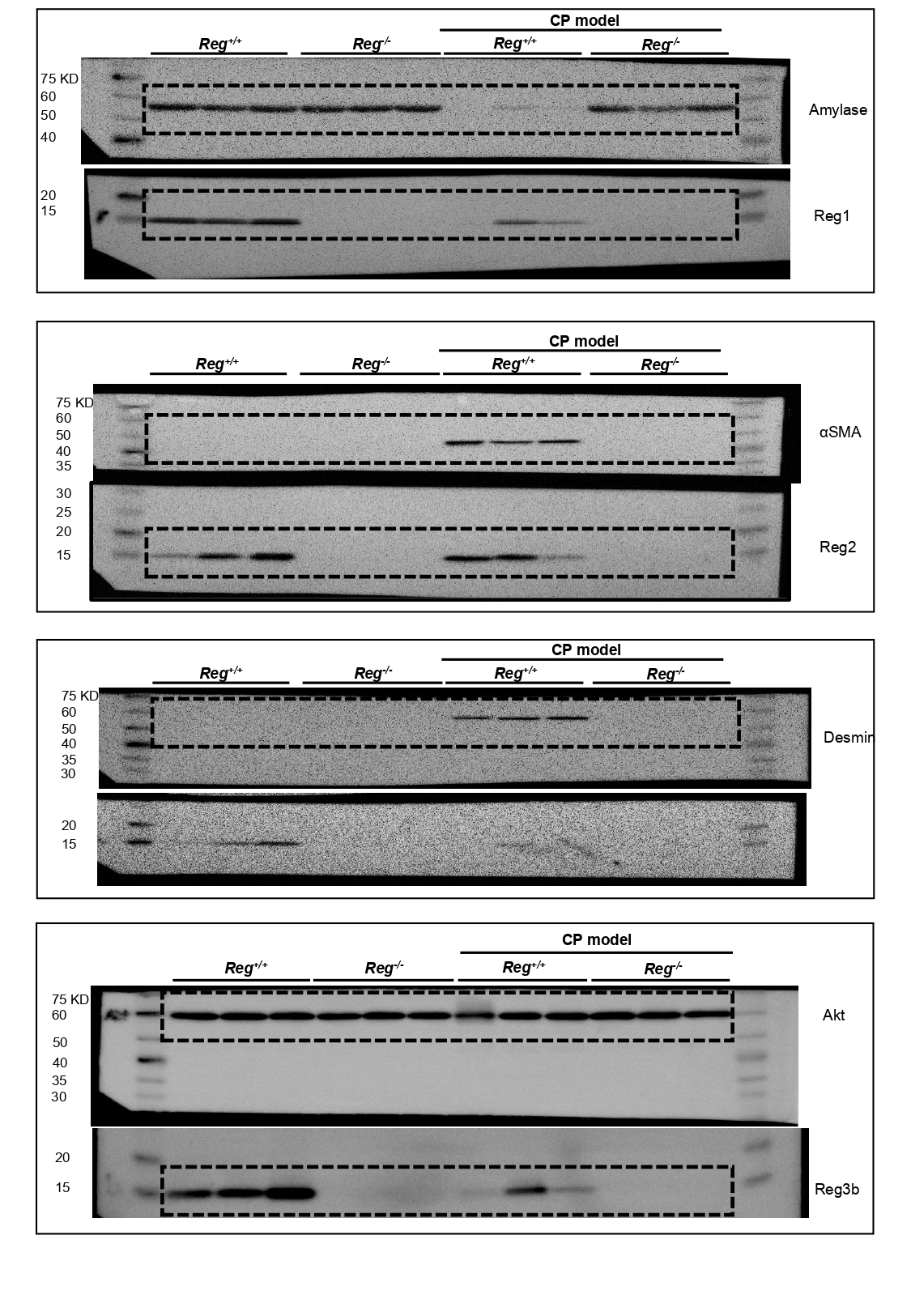


**Supplementary Fig.7** Images of the entire western blots for Fig.5c at 4weeks. Dashed line indicated cropped part of each protein.


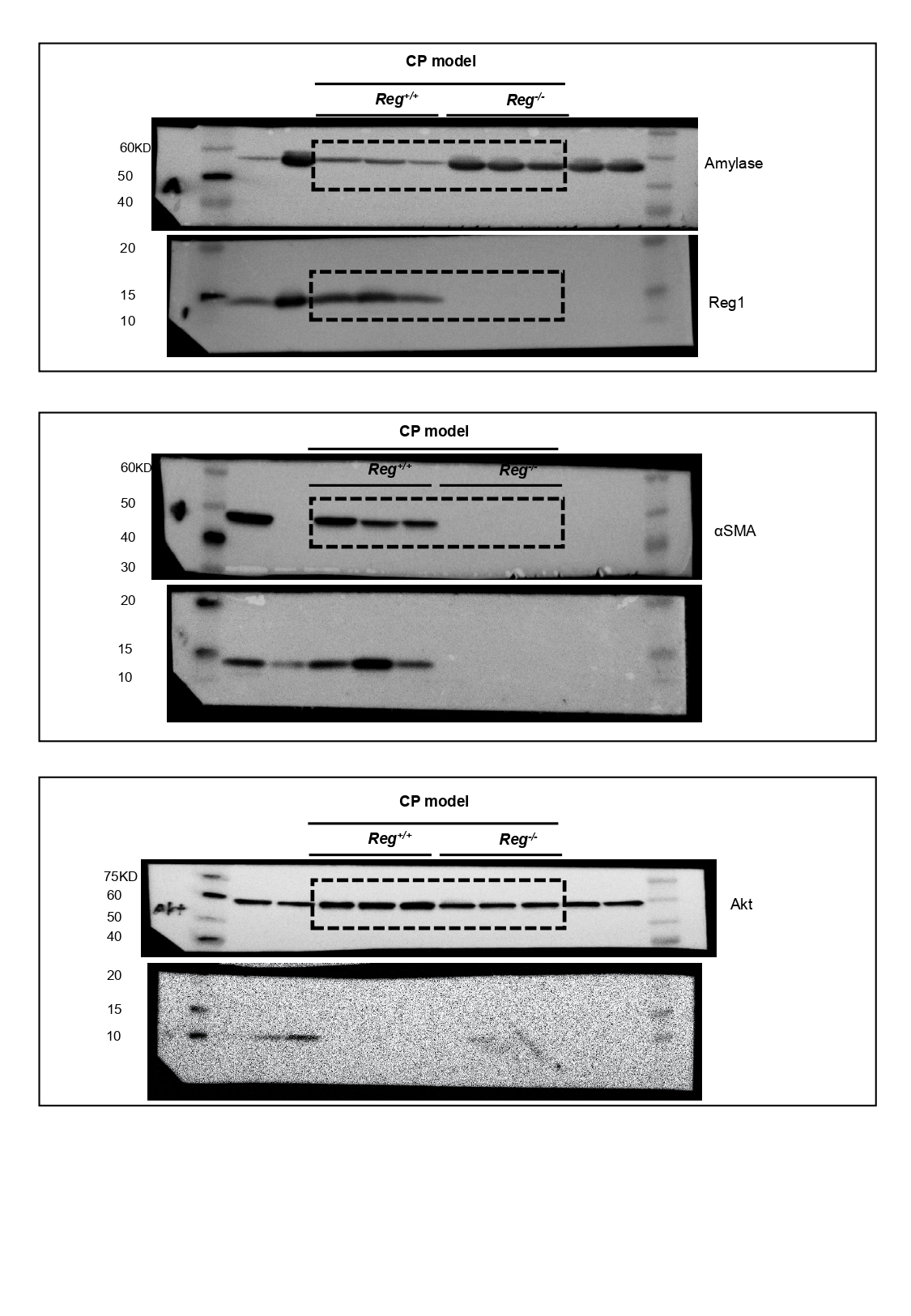


**Supplementary Fig.8** Images of the entire western blots for Fig.6c. Dashed line indicated cropped part of each protein.
